# Novel Approach Sizing and Routing of Wireless Sensor Networks for Applications in Smart Cities

**DOI:** 10.3390/s21144692

**Published:** 2021-07-09

**Authors:** Esteban Inga, Juan Inga, Andres Ortega

**Affiliations:** 1 Postgraduate Department, Smart Grid Research Group (GIREI), Universidad Politécnica Salesiana, Quito 170525, Ecuador; 2 Telecommunications Engineering, Telecommunications Research Group (GITEL), Universidad Politécnica Salesiana, Quito 010102, Ecuador; jinga@ups.edu.ec; 3 Telecommunications Engineering, Center for Studies and Sustainable Development (CEDS), Universidad Tecnológica Ecotec, Guayaquil 092301, Ecuador; aortega@ecotec.edu.ec

**Keywords:** smart metering, internet of things, MST, optimization, routing, smart cities, sizing, wireless sensor networks

## Abstract

Citizens are expected to require the growth of multiple Internet of Things (IoT) -based applications to improve public and private services. According to their concept, smart cities seek to improve the efficiency, reliability, and resilience of these services. Consequently, this paper searches for a new vision for resolving problems related to the quick deployment of a wireless sensor network (WSN) by using a sizing model and considering the capacity and coverage of the concentrators. Additionally, three different routing models of these technology resources are presented as alternatives for each WSN deployment to ensure connectivity between smart meters and hubs required for smart metering. On the other hand, these solutions must reduce costs when this type of wireless communication network is deployed. The present work proposes various optimization models that consider the physical and network layers in order to integrate different wireless communication technologies, thus reducing costs in terms of the minimum number of data aggregation points. Using a heterogeneous wireless network can reduce resource costs and energy consumption in comparison to a single cellular technology, as proposed in previous works. This work proposes a sizing model and three different models for routing wireless networks. In each case, constraints are evaluated and can be associated with different real-world scenarios. This document provides an optimization model that encompasses all of the proposed constraints; due to the combinatorial nature of the problem, this would require a heuristic technique.

## 1. Introduction

This paper outlines the need for rapid deployment of wireless sensors for applications of the Internet of Things (IoT), which are particularly required in smart cities where, in each case, rapid deployment, low costs, and the possibility of using a variety of technological solutions are required [1,2].

IoT applications for smart cities have critical importance; among these applications, some examples include medical assistance for patients with contagious viruses (COVID-19), smart metering of electricity, home delivery of parcels (medicines, food), and “education 4.0”. Consequently, there are areas of opportunity for advanced technologies and techniques that support innovative services with adequate performance to ensure the control and operations of devices with the possibility of wireless communication [3,4]. Furthermore, telecommunication technologies require rapid deployment in order to cover the demand for smart city applications, such as those mentioned above.

Thus, the central offices of local governments require the monitoring and surveillance of sensors placed in previously defined areas or strata of cities to make the operation of services effective, such as traffic management systems, health management (telemedicine), rubbish collection systems, and image recognition systems, among others [5,6].

Therefore, the deployment and routing of a wireless sensor network (WSN) that is responsible for sending and receiving information to the central office must ensure interconnection with each sensor because the information often goes through several sensors before it reaches the central office. This situation indicates that it is necessary to optimize the number of hops in order to reduce energy consumption in the routing process [7].

Most research in this area is related to IoT in the frame of prototypes and various applications with sensors as emerging technologies, but when it is required to scale to services for smart cities, the context changes because it involves the interconnection of multiple sensors installed for various services. Furthermore, information from multiple sensors must be shared with a central office; therefore, the need to determine the size of a wireless sensor network is justified.

Recent research presented possible technologies for specific uses of IoT in smart cities, such as a low-power wide-area network (LPWAN) known as LoRa, which is the type of LPWAN technology that is most often suggested due to its low power consumption and the ease of performing multiple hops. This is a trendy aspect, and it marks the possibility of heterogeneous wireless sensor networks. The present work provides the possibility of promptly using several wireless sensors for IoT applications in smart city services with management from the central offices of a public or private company through optimal sizing and routing [8,9,10].

Thus, in this work, we present a sizing model that minimizes the number of concentrators or data aggregation points (DAPs) in the set of candidate sites where these DAPs can be installed. The poles or peaks of the electrical distribution network that have an approximate height of 10–15 m are assumed as candidate sites. Furthermore, the DAP capacity considers the maximum number of sensors simultaneously connected in each wireless technology’s coverage radius for each DAP. Therefore, this constraint is incorporated into the optimization model.

Previous research showed the routing stage of the resources to be interconnected with the central office of a public or private company can incorporate a heterogeneous wireless sensor network that accommodates multi-hops [11,12].

The proposed scenario considers a series of sensors that can be deployed in an urban area and used in various innovative city applications, such as in fixed rubbish collectors, electric energy meters, drinking water meters, charging centers for electric vehicles, electric energy micro-grids, CO_2_ emission sensors, and vibration sensors in footbridges, among others. Therefore, the principal idea will be to transmit the information from these sensors to the central offices of public or private companies [13,14].

The main contribution of this paper is a novel idea that enables the routing of several IoT sensors in an authentic and geo-referenced scenario in which a heterogeneous wireless sensor network can be deployed for multiple smart city services. This paper describes the scenario and the problem with a mathematical approach. It illustrates simulation results to demonstrate the evaluation of the system with respect to variations in input variables, such as the numbers of DAPs and sensors, DAP capacity, DAP coverage, and capacity of the links in comparison with related work [15].

This article is organized as follows. Section 2 describes the related work. Section 3 describes the problem’s formulation. In Section 4, an analysis of the simulation results is carried out. Finally, we conclude this paper in Section 5.

## 2. Related Work

**Figure 1:**
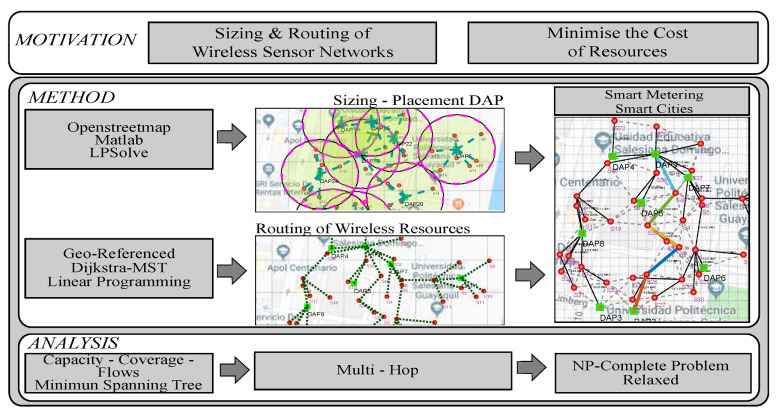
Geo-referenced sizing and routing of wireless sensor networks.

The present work proposes models based on the optimization of the sizing and routing of telecommunications resources in order to achieve connectivity between wireless sensors, concentrators, and the central offices of public or private companies. Figure 1 emphasizes the complete process required for the deployment of a WSN.

Earlier work presented classical wireless sensor network routing solutions, including the novelties of reducing energy consumption with short-range wireless technologies. In addition, hardware prototypes concerned with the interoperability of communication protocols among wireless sensors were presented [4,16].

However, routing can create various logical topologies according to the need for scalability and the use of multi-layers depending on the number of nodes and the physical complexity of the network [17].

There have been contributions to sensor fault location and applications for the reconstruction of a sampled signal with a percentage of measured data through compressed sensing techniques, as presented by [18]. Other contributions sought to improve the quality of service in packet forwarding by using multi-hop delays, and other work addressed the types of wireless sensor networks employed in the IoT. In addition, it is essential to remember that all applications must be scalable, as noted in [19]. There were also contributions to the dimensioning of wireless sensor networks, as presented in [20]. Furthermore, when deploying a wireless network for smart cities, for more sensors to be incorporated daily to generate new services, the WSN must be flexible, and the proposed algorithms must converge to the optimal solution on time for fast management.

The authors note the possibility of using RFID-enabled sensors in an unlicensed band, which can reduce the costs for such applications; however, the interoperability between devices from various manufacturers is not open, and this type of technology was first proposed for electricity metering and for rubbish collection in smart cities.

Other work related to IEEE 802.15.4 (Zigbee) offered alternatives for the achievement of the rapid deployment of a wireless communications network and presented the possibility of implementing network topologies that support various types of topologies, such as star, extended star, or mesh topologies. In addition, other work proposed heterogeneous solutions that sought to ensure that investment in communications infrastructure was minimal. The proposals were hybrid networks based on fiber-optic and wireless networks—called FiWi—or networks based on WiFi and Zigbee [21].

Regarding the technologies for WSNs, data concentrators are gateways, and are also used in LPWAN technologies; therefore, the process of optimal network sizing can also be modeled to adequately locate these concentrators with the same rigor as that in the technologies discussed in the previous paragraph [22]. A routing analysis will determine the technology used in order to interconnect the DAPs required in the WSN’s deployment while considering a star topology among the sensor nodes and gateways.

There are works where the optimization problems were relaxed with clustering, and these were presented in [23,24,25,26,27,28,29].

The optimization problem is of the combinatorial type due to its complexity, and it is called an NP-complete problem. Consequently, some authors proposed heuristic algorithms to explore local optimization solutions that are close to the global solution. On the other hand, there was risk in different works that presented the use of clusters. There were sensors without connectivity in their proposed solutions, even though they previously managed to minimize the cost of energy consumption [23,24]. Methods such as k-means or Kmedoids are not balanced or give multiple answers.

Consequently, other works, such as that of [30], showed the integration of a network from a cross-layer between the access control layer and the routing protocol used to reduce the load of information flow in wireless links. This type of work presents a significant achievement in reducing congested links and, thus, allowing the redirection of data flows in the less saturated network. In this sense, we could consider a multi-layer solution in which the physical layer and the network layer interconnect with each other when sizing and routing the DAPs while taking the wireless link capacity and its coverage radius into consideration.

The need to connect sensors for various applications should not be subject only to the deployment of a unique wireless communication network because using existing technologies reduces the investment cost. Seeking a heterogeneous wireless network solution incorporates the possibility of integrating technologies that facilitate data transmission between sensors and the central office, as is the case of applications linked to the smart metering of electricity, water, or gas.

Graph theory provides an exciting alternative for achieving novel solutions, but above all; it develops solutions with a low computational cost, also facilitates scalability when increasing the number of sensors.

Table 1 refers to the scientific articles related to the relaxation of an NP-complete problem. In addition, the differences between the present work and other proposals are presented.

## 3. Problem Formulation

This work is divided into two sections that offer novel contributions to the rapid implementation of WSNs required for various services in smart cities. This section divides the model into (a) WSN sizing and (b) WSN routing.

### 3.1. Wireless Sensor Network Sizing

For WSN sizing, a square area of *L* by *L* m in an open space is assumed. The data aggregation points (DAPs) will be located in this area to achieve the connectivity of wireless IoT sensors. The DAPs are assumed to enable the connectivity of the sensors within a coverage radius of *R* m; consequently, the wireless sensors can be at any position within the bounded region.

The DAPs can be located at any position within the region and installed on street lighting poles or elevated areas. In this way, the mathematical model minimizes the cost in terms of the lowest number of DAPs. Moreover, the variables used to cover the wireless sensors are described below. A set of *N* sensors is installed in different areas of the region; additionally, we consider a set of *M* possible locations or candidate sites where the are DAPs deployed.

The possible location that was previously described is a candidate for a place where a DAP could be installed or sited; therefore, it will not be mandatory for a DAP to be installed at that location unless it covers a percentage of the sensors. The model defines that a wireless sensor is covered if it is within a distance *R* from at least one DAP; the Haversine distance ($dist_{haversine}$) is used to consider the Earth’s curvature for geo-referenced points [31].

A candidate site is considered an active site if a DAP is enabled or installed on the candidate site. Each DAP has a limited capacity in terms of the sensors. From the above details, an optimization problem is defined that aims to find the minimum number of active sites such that at least a percentage *P* of the sensors are covered.

It is necessary to define a set $S=\{s_{1},s_{2},s_{3},\ldots ,s_{M}\}$ of candidate sites, where the j-th position is given by $\left(xs_{j},ys_{j}\right)$. A set of $D=\{d_{1},d_{2},\ldots ,d_{N}\}$ sensors or wireless devices is also defined. The position of the i-th sensor is given by $\left(xd_{i},yd_{i}\right)$.

We define the quantity $\alpha_{j,i}\in \left\{0,1\right\}$, which implies that if sensor **i** is covered by DAP **j**, then the value is 1; otherwise, the value is 0. Thus, for each candidate site, the quantity $Z_{j}\in \left\{0,1\right\}$ is defined, which implies that the value is 1 when candidate site *j* is an active site.

In the same way, for each sensor $d_{i}$, the quantity $Y_{i}\in \left\{0,1\right\}$ is defined when the value is one, which indicates that the sensor is covered by at least one candidate site. *C* is defined as the capacity of the DAPs to accommodate sensors. The optimization model for the sizing is presented below:

Objective function:

(1) $$\begin{array}{c} \min\;\;\sum_{j=1}^{M}Z_{j}, \end{array}$$

which is subject to:

(2) $$\begin{array}{cc} Y_{i}=\sum_{j=1}^{M}X_{j,i}; & \;\forall \;i\in D; \end{array}$$

(3) $$\begin{array}{cc} \sum_{i=1}^{N}X_{j,i}\leq C\cdot Z_{j}; & \forall \;j\in S; \end{array}$$

(4) $$\begin{array}{cc} \sum_{i=1}^{N}Y_{i}\geq N\cdot P; & \forall \;i\in D; \end{array}$$

(5) $$\begin{array}{cc} X_{j,i}\leq \alpha_{j,i}\cdot Z_{j}; & \forall j\in S;\forall \;i\in D; \end{array}$$

where

- The percentage *P* of sensors are covered in a delimited area or region.
- The term *N* defines the number of sensors in a delimited area or region.
- The term *M* defines the number of candidate sites in a delimited zone or region.
- The number of covered DAPs is $\alpha_{j,i}\in \left\{0,1\right\}$. If a sensor *i* is covered by a DAP *j*, $\alpha_{j,i}$ is 1 and 0 otherwise.
- For each $s_{j}$ candidate site, $Z_{j}\in \left\{0,1\right\}$ is defined, where $Z_{j}$ is 1 if the candidate site is active and 0 otherwise.
- $X_{j,i}$ indicates if sensor *i* is connected to DAP *j*. $X_{j,i}$ is 1 if the connection exists, and 0 otherwise.

Before applying the optimization model that aims to minimize the number of candidate sites for the DAPs, it is necessary to make an on-site visit to verify the availability of the candidate sites for use as inputs for the optimization model.

### 3.2. Wireless Sensor Network Routing

This work suggests three sub-models for the routing of wireless sensor networks with variations that are important to note when planning the deployment of a communication network.

#### 3.2.1. Routing Based on Graph Theory

A set of sensors is defined and connected using one-way wireless communication links. Then, if a link exists between sensor A and sensor B, it is denoted as $e_{A,B}$; in this way, A can send data directly to B. The link $e_{A,B}$ has a weight or distance (Haversine for geo-referencing) that is associated and denoted as $d_{A,B}$.

Here, the concept of graph theory becomes essential, and we define *V* as the set of sensors and *E* as the set of existing links (partial or complete mesh topology). Therefore, graph theory describes G=(V,E) as a directed graph that represents a network topology. Below, a data stream must be transmitted from a source sensor named *S* to a destination sensor called *T*. This flow is transmitted through the intermediate sensors using existing links. The flow between a pair of sensors (s,t) belonging to *V* represents the information’s source and destination.

Then, if we define a path *P* of the set of sensors, we will have that $P=\{P_{1},P_{2},\ldots ,P_{M}\}$ such that the links $e_{Pk,Pk+1}\in E$. Thus, we define the path length *P* as $d_{P}$, which is given by $d_{P}=\sum_{k=1}^{M-1}d_{Pk,Pk+1}$, and define a path for the flow (s,t) as a path $P_{s,t}$ such that $P_{1}=s$ and $P_{M}=t$.

In addition, the path with the minimum distance is defined as the path $P_{s,t^{*}}$ such that $d_{Ps,t^{*}}<=d_{Ps,t}$ for any other possible path $P_{s,t}$. The optimization problem posed in this paper then requires one to find the path with the minimum distance for the flow (s,t).

To write this optimization problem, it is required to define the variable $X_{i,j}$, where the link $e_{i,j}$ is assumed to exist and $X_{i,j}$ has a value of 1 if the link (i,j) belongs to the path $P_{s,t^{*}}$; otherwise, the value is 0. Similarly, for a sensor i∈V, we define $E_{i,out}$ as the set of outgoing links of *i*. We define $E_{i,in}$ as the set of incoming links of *i*.

Objective function:

(6) $$\begin{array}{c} \min\;\;\sum_{(i,j)\in E}d_{i,j}X_{i,j}, \end{array}$$

which is subject to:

(7) $$\begin{array}{cc} \sum_{j|e_{i,j}\in E_{i,out}}X_{i,j}-\sum_{j|e_{i,j}\in E_{i,in}}X_{i,j}=\alpha_{i}; & \;\forall \;i\in V; \end{array}$$

(8) $$\alpha_{i}=\left\{\begin{array}{c} 1,\;\;\mathrm{si}\;i=s\\ -1,\mathrm{si}\;i=t\\ 0,\;\;\mathrm{si}\;i\neq s,i\neq t \end{array}\right.$$

#### 3.2.2. Multicast Routing

This second model proposed for the routing of the wireless sensor network considers a set of sensors connected by bi-directional communication links. For example, if between sensor *A* and sensor *B*, there is a link denoted as $e_{A,B}$, then *A* can send data directly to *B* and *B* can send information directly to *A*. Furthermore, the link $e_{A,B}$ will have a cost, weight, or distance associated with it and is given by $d_{A,B}$.

It is important to note that the link $e_{A,B}$ is arbitrary in the ordering of *a* and *b*; that is, $e_{A,B}$ represents the same as $e_{B,A}$; therefore, the link can be named as $e_{i}$, where *i* is the index of the link.

Now, we define *V* as the set of sensors, *E* as the set of existing links, and G=(V,E) as an undirected graph; additionally, this represents a set of unordered pairs of elements of *V* and, thus, the network topology. An undirected graph indicates that the links are all bidirectional. For this stage, the optimization model seeks to find a minimum-cost spanning tree, which is defined as an undirected graph in which a single path connects any two vertices; that is, a tree is a connected graph with no loops.

Hence, for this problem, we define the set $V=\{V_{1},V_{2},\ldots ,V_{N}\}$ as the set of wireless sensors and we define a tree as a set of links $A=\{e_{1},e_{2},\ldots ,e_{N-1}\}$ such that the links $e_{i}$ ∈ *E*. According to this, the cost of tree *A* can be defined as $d_{A}$ and is represented by $d_{A}=\sum_{i=1}^{N-1}d_{i}$.

Thus, the minimum-cost tree is defined as tree $A^{*}$ such that “$d_{A^{*}}<=d_{A}$” for any other possible tree. The problem started as the need to find a minimum-cost tree, which requires the definition of certain variables, such as $X_{i,j}$, and the establishment of the link $e_{i,j}$ that exists and where $X_{i,j}$ has a value of 1 when the link (i,j) belongs to the solution tree *A*. Otherwise, the value is 0; furthermore, a subset *B* of any sensors belonging to the same set *A* is defined within the group of sensors *V*.

Thus, the problem is defined as the cost minimization of the chosen links and is subject to the constraint that the sensors are connected with N−1 links, where *N* is the number of sensors belonging to *V*.

Objective function:

(9) $$\begin{array}{c} \min\;\;\sum_{(i,j)\in E}d_{i,j}X_{i,j}, \end{array}$$

which is subject to:

(10) $$\begin{array}{c} \sum_{e_{i,j}\in E}X_{i,j}=N-1; \end{array}$$

(11) $$\begin{array}{c} \sum_{e_{i,j}\in E,i\in B,j\in B}X_{i,j}\geq 1;\;\forall \;B\subset V \end{array}$$

#### 3.2.3. Multiple Flow Routing

For the third routing case of the wireless sensor network, a set of sensors is assumed to be connected via unidirectional wireless links. If a link exists between node *A* and node *B*, denoted as $e_{A,B}$, then *A* can send data directly to *B*. The link $e_{A,B}$ has an associated weight or distance and is given by $d_{A,B}$; additionally, the capacity of the link $e_{A,B}$ is given by $C_{A,B}$. Thus, *V* is defined as the set of sensors, *E* as the set of existing links, and G=(V,E) as a directed graph that represents the network topology.

On the other hand, we assume a set of data flows $F=\{f_{1},f_{2},\ldots ,f_{k}\}$ that require the transmission of data from a source node $S_{k}$ to a destination sensor $T_{k}$; this requirement refers to the link capacity in terms of the flow $F_{k}$ and is determined by $R_{k}$. The flow is transmitted through the intermediate sensors by using existing links. A flow between a pair of sensors $f_{k}=\left(S_{k},T_{k}\right)$ belonging to *V* represents the source and destination sensors.

To define the optimization model, it is necessary to define a path *P* as a set of sensors $P=\{P_{1},P2,\ldots ,P_{M}\}$ such that the sensors $e_{Pk,Pk+1}$ ∈ *E*; in addition, we define the length of the path *P* as $d_{P}$, which is given by $d_{P}=\sum_{k=1}^{M-1}d_{Pk,Pk+1}$. We then define the path for the flow (s,t) as a path $P_{s,t}$ such that $P_{1}=s$ and $P_{M}=t$.

The minimum-distance path is defined as the path $P_{s,t^{*}}$ such that $d_{Ps,t^{*}}<=d_{Ps,t}$ for any other possible path $P_{s,t}$. A possible route is defined as a route that contains links that exist within the topology and that can transmit the flows passing through them.

In this way, the optimization problem that seeks to find the minimum-distance path is defined by considering the flows belonging to *F*.

To determine the problem in the field of optimization, it is required to define the variable $X_{i,j,k}$; for this, it is assumed that the link $e_{i,j}$ exists and that $X_{i,j,k}$ has a value of 1 if the link (i,j) belongs to the path $P_{sk,tk}$; that is, the k-th flow uses the link $e_{i,j}$; otherwise, the value is 0.

Similarly, for a sensor i∈V, $E_{i,out}$ is defined as the set of outgoing links of *i* and $E_{i,in}$ is defined as the set of incoming links of *i*.

Overall, the model supporting the above is as follows:

Objective function:

(12) $$\begin{array}{c} \min\;\;\sum_{(i,j)\in E}\sum_{k\in F}d_{i,j}X_{i,j,k}, \end{array}$$

which is subject to:

(13) $$\begin{array}{c} \begin{array}{c} \sum_{j\left|e_{i,j}\in E_{i,out}\right.}X_{i,j,k}-\sum_{j\left|e_{i,j}\in E_{i,in}\right.}X_{i,j,k}=\alpha_{i,k}\\ \forall i\in V;\forall k\in F \end{array} \end{array}$$

(14) $$\begin{array}{c} \sum_{k\in F}R_{k}\cdot X_{i,j,k}\leq C_{i,j};\;\forall \;\left(i,j\right)\in E \end{array}$$

(15) $$\alpha_{i,k}=\left\{\begin{array}{c} 1,\;\;\mathrm{si}\;i=s_{k}\\ -1,\mathrm{si}\;i=t_{k}\\ 0,\;\;\mathrm{si}\;i\neq s_{k},i\neq t_{k} \end{array}\right.$$

In this way, as with multicast routing, one can seek to determine the tree with the minimum cost allowed by the downstream Dijkstra algorithm; however, in this case, it is necessary to subtract the capacity of the links of the current network from the transmission rate of the current flow over the links affected by the same link [32,33].

The pseudocode for the optimal sizing of the wireless sensor network is presented in the ODRSI Algorithm 1, and the pseudocode with the optimal routing according to the sizing results is presented in the OERSI Algorithm 2; furthermore, Table 2 summarizes the variables used in Algorithms 1 and 2.
**Algorithm 1**OSWSN: Sizing of Wireless Sensor NetworksPaso: 1**Definitions****Inputs:** Geo−referencedCoordinatesforDAPsandsensors:$\;\;\;\;\;coordS=\left\{\left(xs_{1},ys_{1}\right),\left(xs_{2},ys_{2}\right),\ldots ,\left(xs_{j},ys_{j}\right),\ldots ,\left(xs_{M},ys_{M}\right)\right\}$;$\;\;\;\;\;coordD=\left\{\left(xd_{1},yd_{1}\right),\left(xd_{2},yd_{2}\right),\ldots ,\left(xd_{i},yd_{i}\right),\ldots ,\left(xd_{N},yd_{N}\right)\right\}$; $C_{j}$, DAPcapacity; *R*, radiusofDAPcoverage; *P*, coveragepercentage;**Output: min**$Z_{j}$;Paso: 2Set coord=coordD∪coordS;Paso: 3Set dist∈$R^{N\times M};\;dist=\;$**0**;**forall** j=1 to M **forall** i=1 to N  $dist\left[i,j\right]=dist_{haversine}\left(coord\left(i\right),coord\left(j\right)\right)$; **endforall****endforall**Paso: 4Apply the optimization model for sizing (Equations (1) to (5));Paso: 5**Return** min:$Z_{j}$;

**Algorithm 2**ORWSN: Routing of Wireless Sensor Networks
Paso: 1
**Definitions**

**Inputs:** dmin=R,

   Geo−referencedcoordinatesforDAPsandsensors:
$\;\;\;\;\;\;coordS=\left\{\left(xs_{1},ys_{1}\right),\left(xs_{2},ys_{2}\right),\ldots ,\left(xs_{j},ys_{j}\right),\ldots ,\left(xs_{M},ys_{M}\right)\right\}$;$\;\;\;\;\;\;coordD=\left\{\left(xd_{1},yd_{1}\right),\left(xd_{2},yd_{2}\right),\ldots ,\left(xd_{i},yd_{i}\right),\ldots ,\left(xd_{N},yd_{N}\right)\right\}$;
**Output:** minimaltreecost
Paso: 2Set coord=coordD∪coordS;Paso: 3Set $G\in R^{\left(N+M\right)\times \left(N+M\right)};\;G=$ **0**;**forall** j=1 to M+N **forall** i=1 to M+N  $G\left[i,j\right]=dist_{haversine}\left(coord\left(i\right),coord\left(j\right)\right)$  **if**  G[i,j]==0;  **then** G[i,j]=∞;  **endif**  **if**  G[i,j]>dmin;  **then** G[i,j]=∞;  **endif**  **if**  G[i,j]≤dmin;  **then** G[i,j]=1;  **endif** **endforall**
**endforall**
Paso: 4
[dp,pred]=dijkstra(G,N+M);
Paso: 5Set path=∅
**forall** i=1:N+M

   node=i;
$\;\;\;path=path\cup \left\{node\right\}$;
**while**  pred(node)<N+M+1 & pred(node)>0

   pred(node);
$\;\;\;path=path\cup \left\{pred(node)\right\}$;$\;\;\;totalCost=totalCost+|dist_{haversine}\left(node,pred\left(node\right)\right)|$;
   node=pred(node);

** endwhile**

**endforall**
Paso: 6**Return**min: routing−tree−path;


## 4. Analysis of Results

The models for solving the sizing and routing of wireless sensor networks required for different smart city applications are presented according to the stages outlined above. The simulation process was performed in Matlab R2020b, which was interfaced with the LPSolve optimizer (developed by MIT) on a computer with an E3-1535M v5 CPU, Intel Xeon 2.90 GHz, and 64 GB of RAM.

### 4.1. Wireless Sensor Network Sizing

The scenario included N=40 sensors located in an approximately *L* × *L* defined area of 300 m${}^{2}$. A total of M=25 candidate sites were established for the placement of a DAP. The optimization model sought to minimize the cost per number of DAPs subject to the capacity constraint of C=20, a coverage radius of R=60 m, and a percentage of P=100%.

**Figure 2:**
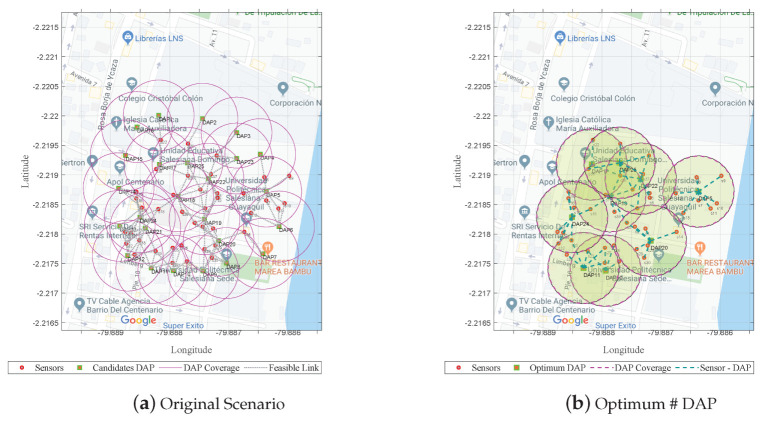
Minimization of the DAPs based on the sizing model.

Figure 2a represents the original scenario before solving the mixed-integer linear programming problem (MILP).

Figure 2b shows that the optimal result was reduced from M=25 to M=9, which would be the sites where the DAPs would be actively placed.

**Figure 3:**
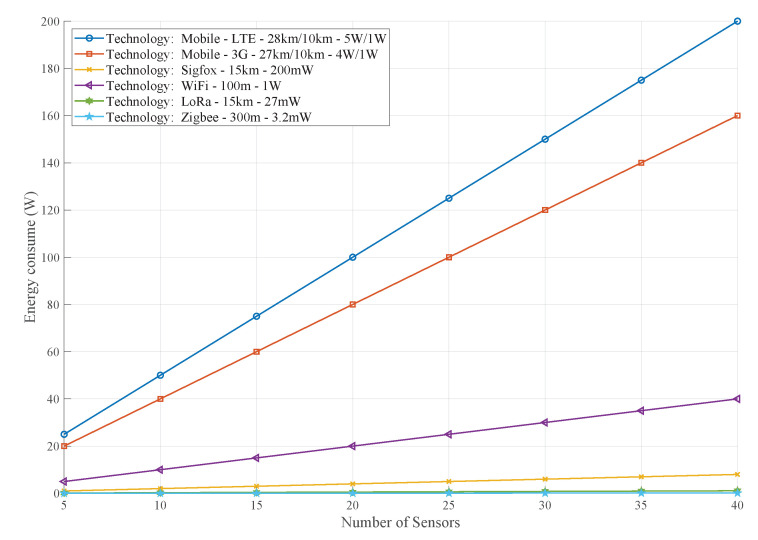
Energy consumption of the wireless technology.

Figure 3 indicates the variations in power consumption among the wireless technologies. It is remarkable to see that when opting for a single technology, such as a cellular network, energy consumption is higher than for technologies that have lower coverage but have a multi-hop option and lower energy consumption.

#### 4.1.1. Routing Based on Graph Theory

After solving the sizing problem, a change in the scenario was generated, and we then had N=40 sensors and M=9 DAPs with which we proceeded to apply the routing model based on graph theory. For this stage, a connectivity matrix was generated among all resources (sensors and DAPs) based on the calculation of distances between two geographical points using the Haversine distance; then, the connectivity matrix *G* was generated based on the passing weight, which was the coverage radius of the DAPs: R=60 m.

**Figure 4:**
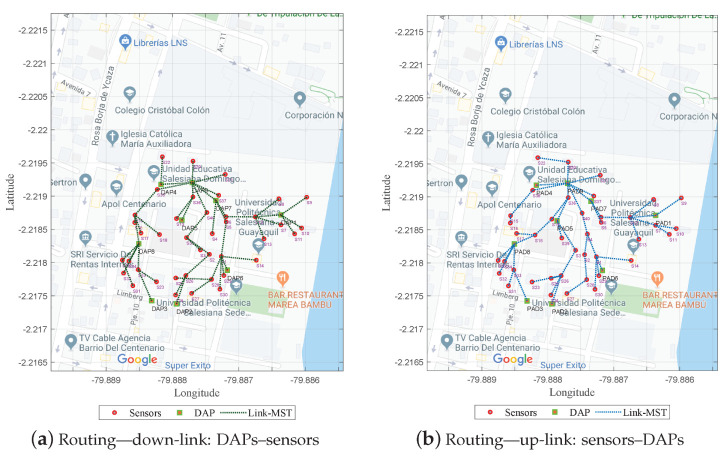
Optimal routing based on the Dijkstra algorithm.

Therefore, to find the minimum cost of the tree, Dijkstra’s algorithm was used. The model presented two minimum-cost trees from two Dijkstra variations. Figure 4a shows the solution generated in the shortest time (0.4219 s) by starting the routing from the DAPs to the sensors (down-link).

Figure 4b presents the routing from the sensors to the DAPs (up-link) in a longer time (1.6094 s). This variation in time and computational performance is important when generating a planning model with a larger number of resources to explore.

#### 4.1.2. Multi-Cast Routing

**Figure 5:**
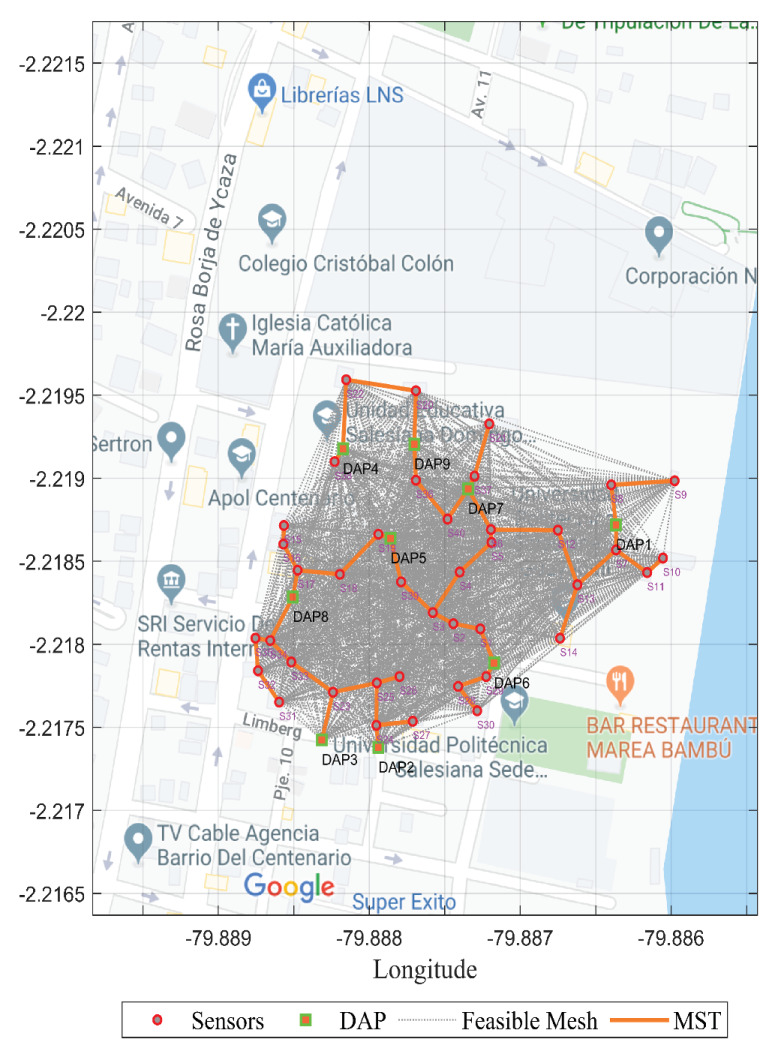
Minimal spanning tree—wireless network backup.

A variation of the routing presented in this work allowed us to make a minimum spanning tree (MST) to link the resources to be placed (sensors and DAPs). This offered the possibility of achieving an MST that generated resilience when interconnecting the resources. This solution is less expensive than a partial or total mesh topology in smart cities depending on the type of application. If it is necessary to ensure the real-time collection of information, this type of routing can contribute. Figure 5 presents a feasible mesh from the distance restriction represented by the coverage radius (R=60 m); it depends on this variable. The MST was determined to generate a multi-cast routing.

#### 4.1.3. Multiple-Flow Routing

**Figure 6:**
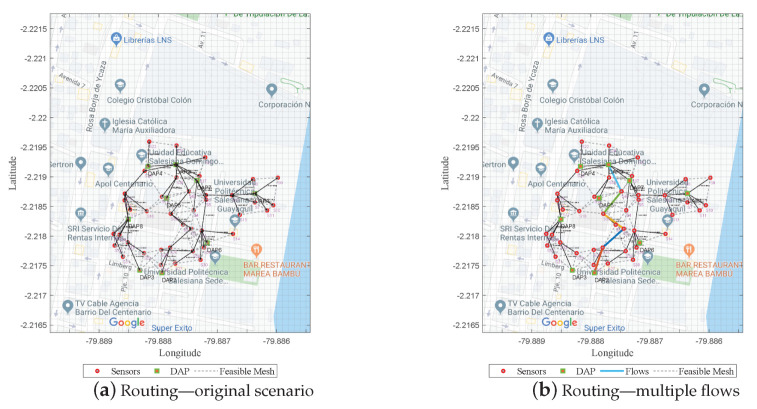
Optimal routing of wireless link—capacity link constraint.

In general, this model presents a routing that considers the capacity of the links as a constraint. In addition, it calculates the number of data flows that can pass through each wireless link; in the case of finding a congested route, the model shows resilience and evaluates another route through which it can send the data. Figure 6a shows the MST achieved in the previous stage, which was acted upon by defining links with an origin and destination; the cost and link capacity were previously use to make a flow matrix with its source and destination, as well as a requirement in terms of the number of packets to be sent. Figure 6b presents the best route that was constructed by the algorithm, which connected the link from DAP9 to DAP2.

**Figure 7:**
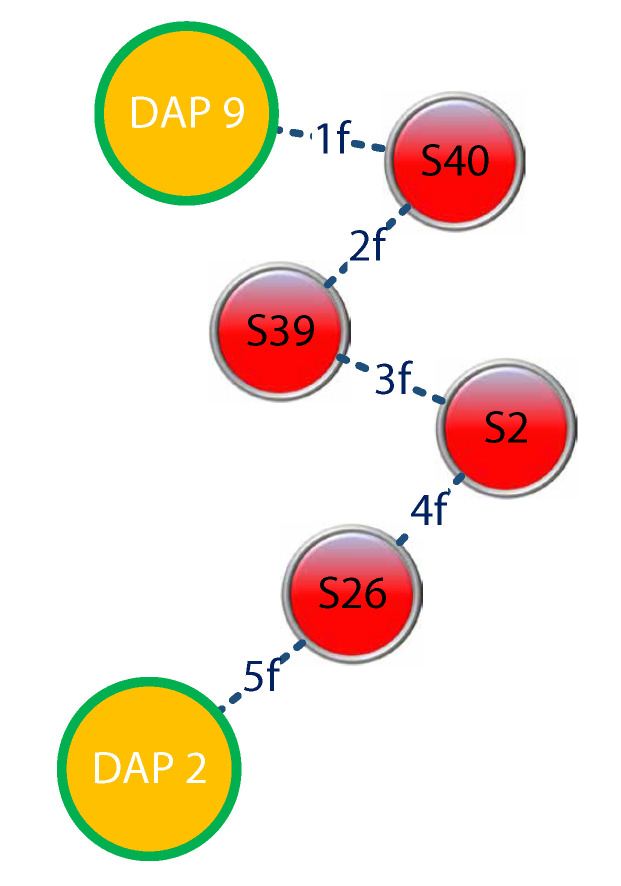
Routing with the capacity of the links—Table 3.

In Figure 7, we can see that the most significant amount of flow per wireless link was from DAP2 due to the number of DAPs around it. It was solved as an MILP.

This model allowed us to dimension the amount of traffic that could circulate from the beginning to the end in order to optimize the resources and ensure the correct quality of service in the wireless links. Table 3 shows the results generated by LPSolve.

**Figure 8:**
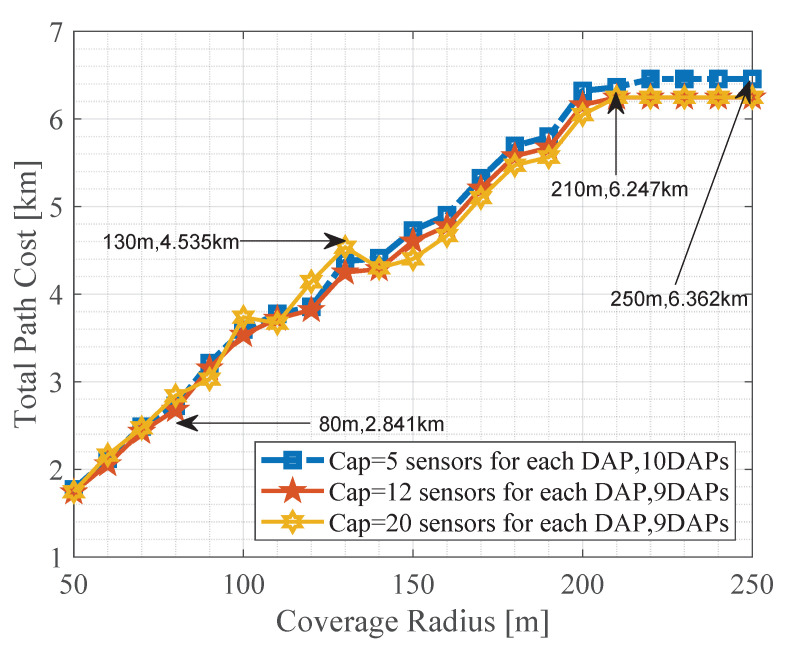
Coverage radius vs. total path cost.

The capacity of each DAP has a direct impact on the search for optimal candidate sites and the problem graph because the selected DAPs may be different. Therefore, there is a direct impact on the total path cost when varying the capacity of a DAP. However, when the capacity of each DAP is low, the total path cost is higher and may remain somewhat higher in contrast to medium or higher capacities. Figure 8 also shows that there is a trend of linear growth as the the radius of coverage becomes more significant. Depending on the arrangement of the resulting networks, scenarios with higher total path costs can occur. Finally, Figure 8 shows that after 200 m of coverage, the total path cost remains constant.

Table 4 identifies the innovations of this research in contrast to those of other proposals that address the problem of the sizing and routing of wireless sensors. The present work focuses on scenarios such as the smart metering of electrical energy; in fact, it focuses on relaxing the complexity of the sizing and routing problem by independently and consecutively solving each stage. In this way, the present work was compared with other proposals to highlight the contributions regarding the problem addressed.

## 5. Conclusions

The model for the resolution of the problem of flow capacity achieved the optimal coordination of data routing in the network layer and allocated resources in the physical layer by fixing the costs in the capacities of the DAPs, thus providing a cross-layer solution.

The novel contribution of this model was the establishment of a multi-hop WSN that used cross-layer information to determine the routing of the network layers while involving decision aspects, such as wireless link capacity and traffic flow demands. The model did not use clustering methods to relax the NP-complete problem.

This paper focused on the sizing and routing of wireless sensor networks deployed for the provision of new IoT services and applications for smart cities by considering the locations of DAPs in a neighborhood area network.

Therefore, to achieve this objective, a problem was formulated in three stages (graph theory, multi-casting, and multiple flows) that were sequentially simulated, but had the same goal: to minimize the cost of the minimum number of DAPs and the minimum distance.

This paper presented an actual urban area that was geo-referenced with the latitude and longitude of each technological resource, and the performance of the optimization models was evaluated. Simulation results verified that the proposed solution could reduce the number of DAPs and generate random answers based on clustering methods. Additionally, a minimum-cost tree was initially created by considering the weight of the cost per distance for the backup wireless network. Finally, the binding capacity restriction was added.

Inter-DAP interference was not considered in this work, as it can be solved by assigning different channels to each DAP. 

## Figures and Tables

**Table 1:** Summary of papers related to the sizing and geo-referenced routing of wireless sensor networks.

Scientific Paper	Problem				Constraints				Proposal			
**Author**	**Energy Efficiency**	**Data Collection**	**Scalability**	**DAP** **Placement**	**Multi-Hop**	**Capacity**	**Coverage**	**Cost**	**Clustering Conglomerate**	**GIS**	**Energy** **Consumption**	**Cross-Layer**
Wang et al. [23]	✠	✠		✠	✠		✠	✠	✠	✠	✠	
Hassan [24]	✠	✠		✠	✠		✠	✠	✠	✠	✠	
Wang et al. [25]	✠	✠		✠	✠		✠	✠	✠	✠	✠	
Guodong [26]	✠	✠		✠	✠		✠	✠	✠	✠	✠	
Wang et al. [27]	✠	✠		✠	✠		✠	✠	✠	✠	✠	
Passos [7]	✠	✠	✠	✠	✠	✠	✠	✠	✠		✠	
Masoud [6]	✠	✠			✠	✠	✠	✠	✠		✠	
Afaneh [2]	✠	✠	✠	✠	✠		✠	✠		✠	✠	
Wang [29]	✠	✠			✠		✠	✠	✠		✠	
Current Work		✠	✠	✠	✠	✠	✠	✠	✠	✠		✠

**Table 2:** Variables of Algorithms 1 and 2.

Variable	Definition
$C_{j}$	Capacity of DAP
*R*	Coverage radius of wireless technology/DAP
*P*	Wireless coverage percentage
dist	Distance matrix: from each sensor to each candidate site
coordS	Coordinates of sites for DAPs
coordD	Coordinates of sensors
*M*	Number of candidate sites
*N*	Number of sensors
$Z_{j}$	Set of links
$X_{j,i}$	Wireless link
$Y_{i}$	Sensor with coverage of a DAP
dmin	Minimum distance of wireless technology
*G*	Connectivity matrix—graph
dp	Minimum distance between resources and a vertex
pred	Vertex preceding v in the shortest path
path	Connectivity path
totalCost	Tree extension in meters

**Table 3:** ZigBee simulation (250 Kbps)—flows were generated by using LPSolve.

Source Node	Destination Node	Requirement—# of Flows	Link Cost (Kbps)	Link Capacity—# of Flows	MILP—# of Flows
49	40	1	230	10	1
40	39	1	240	10	2
39	2	1	247	10	3
2	26	1	250	10	4
26	42	1	248	10	5

**Table 4:** Summary: the main contributions of similar research.

Goal	Proposal	A1 [26]	A2 [23]	A3 [25]	A4 [24]	A5 [27]
**Sizing**						
DAP location	Candidate sites	Random	Random	Random	Random	Random
Haversine distance	✓	✓	✗	✓	✓	✗
Euclidean distance	✗	✗	✓	✗	✗	✗
Optimization MILP	✓	✗	✗	✗	✗	✗
K-means clustering	✗	✓	✓	✓	✓	✓
Coverage	✓	✓	✓	✓	✓	✓
DAP capacity	✓	✗	✗	✗	✗	✗
**Routing**						
Shortest path G=(V,E)	Dijkstra $O(n^{2})$	Floy Warshall $O(|V{|}^{3})$	Floy Warshall $O(|V{|}^{3})$	Floy Warshall $O(|V{|}^{3})$	✗	✗
Backup minimum spanning tree + multi-hops	PRIM O(Elog(V))	✗	✗	✗	✗	✗
Shortest path G=(V,E) + link capacity + Weight (bps)	✓	✗	✗	✗	✗	✗

## Data Availability

Not applicable.

## References

[1] Khalil M., Khalid A., Khan F.U., Shabbir A. A review of routing protocol selection for wireless sensor networks in smart cities. Proceedings of the 24th Asia-Pacific Conference on Communications, APCC.

[2] Afaneh A., Shahrour I. (2017). Use of GIS for SunRise Smart City project, large scale demonstrator of the Smart City. SENSET.

[3] Jain B., Brar G., Malhotra J., Rani S. (2017). A novel approach for smart cities in convergence to wireless sensor networks. Sustain. Cities Soc..

[4] S S.N., Mane P.B. (2017). Swarm Intelligent WSN for Smart City. Proc. Int. Conf. Data Eng. Commun. Technol..

[5] Jawhar I., Mohamed N., Al-Jaroodi J. (2018). Networking architectures and protocols for smart city systems. J. Internet Serv. Appl..

[6] Masoud M.Z., Jaradat Y., Jannoud I., Al Sibahee M.A. (2019). A hybrid clustering routing protocol based on machine learning and graph theory for energy conservation and hole detection in wireless sensor network. Int. J. Distrib. Sens. Netw..

[7] Passos D., Rolim G., Ribeiro I., Moraes I., Albuquerque C. (2019). Robust Advanced Metering Infrastructures and Networks for Smart Grid.

[8] Hanif S., Khedr A.M., Aghbari Z.A., Agrawal D.P. (2018). Opportunistically Exploiting Internet of Things for Wireless Sensor Network Routing in Smart Cities. J. Sens. Actuator Netw..

[9] Kumar D., Aseri T.C., Patel R.B. (2011). EECDA: Energy efficient clustering and data aggregation protocol for heterogeneous wireless sensor networks. Int. J. Comput. Commun. Control.

[10] Dabirmoghaddam A., Ghaderi M., Williamson C. (2014). On the optimal randomized clustering in distributed sensor networks. Comput. Netw..

[11] Meenaakshi Sundhari R.P., Jaikumar K. (2020). IoT assisted Hierarchical Computation Strategic Making (HCSM) and Dynamic Stochastic Optimization Technique (DSOT) for energy optimization in wireless sensor networks for smart city monitoring. Comput. Commun..

[12] Senthilkumar R., Tamilselvan G.M., Kanithan S., Arun Vignesh N. (2019). Routing in WSNs powered by a hybrid energy storage system through a CEAR protocol based on cost welfare and route score metric. Int. J. Comput. Commun. Control.

[13] Abujubbeh M., Al-Turjman F., Fahrioglu M. (2019). Software-defined wireless sensor networks in smart grids: An overview. Sustain. Cities Soc..

[14] Kumar D., Aseri T.C., Patel R.B. (2011). A novel multihop energy efficient heterogeneous clustered scheme for wireless sensor networks. Tamkang J. Sci. Eng..

[15] Wang W. (2020). Deployment and optimization of wireless network node deployment and optimization in smart cities. Comput. Commun..

[16] Hidalgo Lopez R., Moreno Novella J.I. (2011). Routing Design in Wireless Sensor Networks and a Solution for Healthcare Environments. IEEE Lat. Am. Trans..

[17] Guidoni D.L., Souza F.S., Ueyama J., Villas L.A. (2014). RouT: A routing protocol based on topologies for heterogeneous wireless sensor networks. IEEE Lat. Am. Trans..

[18] Inga-ortega J., Inga-ortega E., Gómez C. Electrical Load Curve Reconstruction required for Demand Response using Compressed Sensing Techniques. Proceedings of the IEEE PES Innovative Smart Grid Technologies Conference—Latin America (ISGT Latin America).

[19] Inga E., Céspedes S., Hincapié R., Cárdenas A. (2017). Scalable Route Map for Advanced Metering Infrastructure Based on Optimal Routing of Wireless Heterogeneous Networks. IEEE Wirel. Commun..

[20] Inga E., Eléctrica I., Campaña M., Eléctrica I., Hincapié R., Céspedes S. Optimal Placement of Data Aggregation Points for Smart Metering using Wireless Heterogeneous Networks. Proceedings of the 2018 IEEE Colombian Conference on Communications and Computing (COLCOM).

[21] Peralta A., Inga E., Hincapié R. (2017). Optimal Scalability of FiWi Networks Based on Multistage Stochastic Programming and Policies. J. Opt. Commun. Netw..

[22] Mekki K., Bajic E., Chaxel F., Meyer F. Overview of cellular LPWAN technologies for IoT deployment: Sigfox, LoRaWAN, and NB-IoT. Proceedings of the 2018 IEEE International Conference on Pervasive Computing and Communications Workshops (Percom Workshops).

[23] Wang G., Zhao Y., Ying Y., Huang J., Winter R.M. (2018). Data Aggregation Point Placement Problem in Neighborhood Area Networks of Smart Grid. Mob. Netw. Appl..

[24] Hassan A., Zhao Y., Pu L., Wang G., Sun H., Winter R.M. Evaluation of Clustering Algorithms for DAP Placement in Wireless Smart Meter Network. Proceedings of the 2017 9th International Conference on Modelling, Identification and Control (ICMIC).

[25] Wang G., Zhao Y., Huang J., Winter R.M. On the Data Aggregation Point Placement in Smart Meter Networks. Proceedings of the 2017 26th International Conference on Computer Communication and Networks (ICCCN).

[26] B G.W., Zhao Y., Ying Y., Huang J., Winter R.M. (2018). A Clustering Algorithm for the DAP Placement Problem in Smart Grid. Adv. Hybrid Inf. Process..

[27] Wang G., Zhao Y., Huang J., Duan Q., Li J. A K-means-based Network Partition Algorithm for Controller Placement in Software Defined Network. Proceedings of the 2016 IEEE International Conference on Communications (ICC).

[28] Janani E.S.V., Univesity A. Analytical techniques to characterize and optimize the performance of sensor network systems. Proceedings of the IEEE- Fourth International Conference on Advanced Computing, ICoAC 2012 MIT.

[29] Wang J., Gao Y., Liu W., Sangaiah A.K., Kim H.J. (2019). Energy efficient routing algorithm with mobile sink support for wireless sensor networks. Sensors.

[30] Inga E., Hincapié R., Céspedes S. (2019). Capacitated Multicommodity Flow Problem for Heterogeneous Smart Electricity Metering Communications Using Column Generation. Energies.

[31] Winarno E., Hadikurniawati W., Rosso R.N. Location based service for presence system using haversine method. Proceedings of the 2017 International Conference on Innovative and Creative Information Technology (ICITech).

[32] Dijkstra E.W. (1959). A note on two problems in connexion with graphs. Numer. Math..

[33] Johnson D.B. (1973). A note on Dijkstra’s shortest path algorithm. J. ACM (JACM).

